# A causal relationship between functional connectivity of brain networks and cardiovascular disease: A Mendelian randomization study

**DOI:** 10.1097/MD.0000000000043131

**Published:** 2025-07-04

**Authors:** Haoxuan Chu, Xia Guo, Han Chi Xu, Shipeng Wang, Zhen Guo, Yulin Tian, Yushi Wang

**Affiliations:** a Department of Cardiovascular Medicine, The First Hospital of Jilin University, Changchun, China.

**Keywords:** brain network function, cardiovascular disease, causal inference, Mendelian randomization, nutrition

## Abstract

Dysfunction in the brain’s resting-state functional networks is strongly connected with mental illness and cognitive impairment, while cardiovascular disease (CVD) is accepted as a risk factor for cognitive dysfunction. Growing interest exists in the correlation between brain and heart diseases. However, the causality between resting-state functional networks and CVD remains uncertain. In this research, a two-sample Mendelian randomization (MR) approach was applied to explore the causal association between 191 resting-state functional magnetic resonance imaging (rsfMRI) traits and 10 CVDs. This MR study employed single nucleotide polymorphisms that are strongly associated with rsfMRI phenotypes, which were sourced from the Zenodo database. We aimed to determine the causal relationship between rsfMRI phenotypes, considered as the exposure, and CVD, defined as the outcome. We engaged inverse variance weighting as our primary analytical method and executed a comprehensive sensitivity analysis to assess the heterogeneity and dependability of the results. Additionally, to validate the robustness of the findings, the test thresholds were recalibrated using the Bonferroni correction method. Functional connectivity among the angular gyrus, precuneus, cingulate gyrus, and parietal lobe also increased the hazards of atrial fibrillation (OR_IVW_ = 0.644, 95% CI: 0.527–0.788, *P* = 1.83 × 10^‐5^). Moreover, MR analyses of brain network connectivity concerning other CVDs did not meet the Bonferroni-corrected *P*-value threshold. Changes in functional connectivity of brain networks may be an indicator of risk for the development of atrial fibrillation but are not associated with the development of other CVDs.

## 1. Introduction

Cardiovascular disorders (CVDs) represent the foremost contributors to global disease burden and fatal outcomes. The prevalence of CVDs has risen dramatically in the past several decades, increasing from 31.31 million cases in 1990 to 55.45 million cases in 2019, representing a rise of 77.12%, highlighting the urgent need for effective prevention and treatment strategies.^[1]^ Common risk factors for CVD include hyperlipidemia, diabetes mellitus, obesity, and smoking, which also contribute to structural brain abnormalities.^[2,3]^ The brain is also a complex organ with distinct subdivisions, each responsible for specific functions. These subdivisions are interconnected, forming networks that mediate various cognitive, affective, and behavioral processes. Furthermore, there is not a one-to-one correspondence between the anatomy and physiology of the brain.^[4]^ Artery-brain and heart-brain circuits make the cardiovascular system accessible to the brain through multiple layers of afferent and efferent polysynaptic axons, and these circuits allow for the integration of cardiovascular system signals into the brain, which affects a variety of brain functions and potentially influence the progression of CVD.^[5]^

Several studies have described the correlation between functional brain networks and CVD. Patients with heart failure (HF) may exhibit reduced resting-state functional connectivity (FC) between the caudate nucleus and pontine cerebellar regions in the right hemisphere, as well as increased FC in regions such as the middle frontal gyrus and sensorimotor regions, which are thought to be associated with cognitive and emotional function.^[6]^ Hypertension, alternatively, causes alterations in FC patterns in key brain regions including the cerebellum and prefrontal cortex.^[7]^ In addition, previous studies indicate that neurogenic cardiac damage may emerge after acute ischemic stroke thereby allowing an increased incidence of arrhythmias, hypertension, and myocardial infarction.^[8,9]^ Resting-state functional magnetic resonance imaging (rsfMRI) is a noninvasive neuroimaging method that is employed to measure the functional activity of the brain in a resting state. This technique enables the detection of spontaneous neural activity and FC in different regions of the brain in a resting state, and it is commonly used to study functional brain networks.^[10]^ However, no study has yet demonstrated causality between the reticular formation of the brain and CVD, possibly because prior studies have been confounded by reverse causation and confounding factors. Mendelian randomization (MR) is a method of inferring causality using genetic variants strongly correlated with exposure as a tool.^[11]^ As alleles are randomly assigned, MR reduces confounding effects and the interference of reverse causality.^[12]^ Thus, we performed a two-sample MR study to evaluate the causal association between CVD and brain network function.

## 2. Materials and methods

### 2.1. Study design

**Figure 1.**
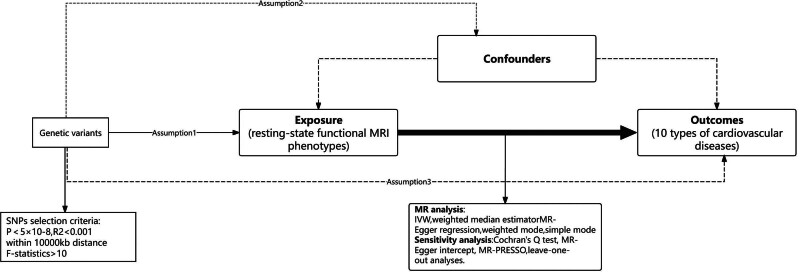
Overall experimental design. Assumption1, instrument variables are robustly related to exposure; Assumption2, instrument variables are not related to confounders; Assumption3, instrument variables are related to outcome only through exposure. IVW = inverse variance weighted, MR = Mendelian randomization, MRI = magnetic resonance imaging, SNPs = single-nucleotide polymorphisms.

A two-sample MR analysis was conducted using data from a genomic association study to evaluate the causality between functional brain networks and CVDs. For the reliability of the study, our study must satisfy the 3 main assumptions of MR: 1. Correlation hypothesis: instrumental variables (IVs) were required to significantly correlated with exposure (brain network function). The strength of IVs was ensured in this study by strict genome-wide association study (GWAS) thresholds (*P* < 5 × 10^‐8^) and linkage disequilibrium screening. 2. Independence hypothesis: IVs should not be associated with confounders. We excluded IVs associated with known confounders by PhenoScanner. 3. Exclusivity assumption: single-nucleotide polymorphisms (SNPs) affect outcomes only through exposure and not through other biological pathways. Horizontal pleiotropy is the main risk of violating this assumption. We tested and corrected for such biases by MR-Egger regression and MR-PRESSO^[13]^ (Fig. 1).

### 2.2. GWAS data sources for brain network function

Genetic instruments related to brain network function were obtained from an analysis of rsfMRI data across 4 distinct datasets, encompassing a total of 44,190 participants. The study used Independent Component Analysis to ascertain functional regions of the brain and generate 1777 phenotypes for exploring the genetic framework of brain functional activity, and GWAS methods in 34,691 individuals of British ancestry to identify and find that many of the genetic variants associated with brain functional activity are significantly linked with cognitive, psychiatric disorders (e.g., schizophrenia, depression) as well as significant genetic sharing of structural brain features.^[14]^ Given that genetic variants exhibit weaker effects on brain functional networks relative to structural features, this MR analysis included only 191 traits showing genome-wide significant associations (*P* < 5 × 10^‐8^),^[15,16]^ which consisted of 111 pairwise FC (edges), 75 amplitude traits (nodes) representing regional spontaneous neural activity, and 5 global FC.

### 2.3. GWAS data sources for CVDs

To minimize sample overlap between exposures and outcomes, we obtained CVD statistics from GWAS results from FinnGen. The FinnGen project is a research initiative that integrates genomic data with digital health information intending to use genetic research to identify new therapeutic targets and diagnostics for the treatment of a wide range of diseases. To date, FinnGen has successfully collected genomic data from over 400,000 Finnish participants. Detailed information on various CVDs is detailed in Table 1.

**Table 1:** Overview of cardiovascular diseases included in this study.

Cardiovascular diseases	Abbreviation	Sample size	Ancestry
Atrial fibrillation	AF	50,743 cases and 210,652 controls	EUR
Coronary heart disease	CHD	46,959 cases and 365,222 controls	EUR
Diabetes	–	42,593 cases and 337,038 controls	EUR
Heart failure	HF	29,218 cases and 381,838 controls	EUR
Hypertrophic cardiomyopathy	HCM	553 cases and 312,154 controls	EUR
Ischaemic heart disease	IHD	69,008 cases and 343,173 controls	EUR
Peripheral vascular diseases	PVD	2489 cases and 381,977 controls	EUR
Restrictive cardiomyopathy	RCM	99 cases and 405,843 controls	EUR
Rheumatic heart valve disease	RHVD	741 cases and 411,147 controls	EUR
Stroke	–	43,132 cases and 297,867 controls	EUR

AF = atrial fibrillation.

### 2.4. Instrument selection

During the study, SNPS that are closely related to exposure factors need to be identified. To establish methodological robustness in delineating neurocircuitry-cardiovascular pathophysiology associations, rigorous selection protocols were implemented for IV specification. SNPs were required to be highly correlated with the exposure factors and significant in GWAS, with a *P*-value of less than 5 × 10^‐8^. To verify the independence of individual SNPs, the genetic distance was set to 10,000 kb, and to avoid interference caused by linkage disequilibrium, SNPs with *R*^2^ > 0.001 were removed within 10,000 kb. When palindromic alleles were present, allele frequencies were used to infer alleles of the forward strand. To circumvent potential bias resulting from weak IVs, we will calculate the *F* statistic for each SNP to ascertain the strength of the correlation. The *F* statistic is calculated as: *F* = *R*^2^ × (n‐*k*‐1) ÷ [*k* × (1‐*R*^2^)], where *R*^2^ denotes the proportion of variation explained by the IVs to the exposure, N represents the sample size, and *k* indicates the number of IVs. The size of the exposure dataset, and *K* is the number of SNPs. The statistical significance of the SNPs was evaluated by an *F*-statistic threshold of 10.^[17]^ The *R*^2^ value is determined as follows: *R*^2^ = 2 × (1‐EAF) × EAF × (β/SD)^2^, where MAF is the minor allele frequency, *β* is the allele effect value, and SD is the standard deviation. A value exceeding the threshold indicates that the SNP is statistically robust. Otherwise, it is not deemed sufficiently robust and is excluded from further study. Building upon established evidence regarding modifiable risk factors for cardiovascular disorders and neural network alterations (encompassing hypertensive status, alcohol consumption, smoking habit), we used PhenoScanner to remove promiscuous SNPS and performed strict quality control.^[18–21]^ Finally, we filtered SNPS that showed significant genome-wide associations (*P* < 5 × 10^−8^) with these predetermined covariates^[22]^ (Table S1, Supplemental Digital Content, https://links.lww.com/MD/P311).

### 2.5. Statistical analysis

All statistical analyses were performed using R software (version 4.4.0). Inverse variance weighting (IVW) estimation was the main statistical method. The IVW technique is founded upon the tenet of ascribing greater weights to data points exhibiting lower variability, thereby enhancing the precision and robustness of the analysis, particularly in the estimation of IVs. The statistical advantage of the IVW technique lies in its ability to integrate information from disparate studies through weighting, thereby effectively estimating the true effect size.^[23]^ The weighted median employs a weighting system that considers the strength and validity of the IVs, effectively accounting for the potential influence of a null instrument. This approach offers robust estimates of causal effects, even when as much as 50% of the data comes from genetic variations with invalid IVs.^[24]^ To strengthen methodological rigor and result consistency in MR analyses, we implemented a comprehensive sensitivity validation framework encompassing Cochran Q statistics, pleiotropy assessments, and leave-one-out iterations. The Cochrane Q test was employed to conduct a heterogeneity analysis. The test statistic Q is calculated by comparing the observed proportional variability between groups with the expected variability under the null hypothesis. This allows for the measurement of the extent of between-group variability relative to total variability.^[25]^ The MR-PRESSO method and the MR-Egger regression intercept test were employed to identify horizontal pleiotropy, a potential source of bias in MR studies. MR-Egger regression addresses horizontal pleiotropic bias through an intercept-based weighted linear regression framework. In this method, genetic association estimates between IVs and the outcome are regressed on their corresponding exposure effect estimates, with inverse variance weighting applied to account for estimation precision. The intercept term quantifies the average pleiotropic effect across IVs, where a statistically significant deviation from zero (*P* < .05) indicates directional pleiotropy. This methodology effectively mitigates systematic pleiotropic bias. However, its accuracy is contingent upon sufficient instrument strength and sample size, and it remains vulnerable to bias amplification under weak instrument conditions.^[26–28]^ MR-PRESSO first identifies genetic IVs that may be polytomous through outlier detection, followed by a global polytomous test, which compares the residual sums of squares of the models before and after the removal of the outliers, and indicates the presence of horizontal polytomousness if the difference is significant (*P* < .05). Ultimately, MR-PRESSO improves the reliability of the results by reestimating the effect sizes by removing outliers. This method does not rely on assumptions in the direction of polytropy, which is superior to the limitations of MR-Egger, but requires sufficient IVs to ensure validity and usually needs to be combined with other methods to verify robustness.^[29]^ Both methods assess horizontal pleiotropy in MR studies by evaluating the regression intercept. MR-PRESSO is primarily utilized to identify and correct for outliers in genetic instruments, whereas MR-Egger regression directly estimates the mean pleiotropy effect.^[29,30]^ The leave-one-out sensitivity procedure iteratively excludes individual SNPs from the IV set to assess the potential overrepresentation of any particular genetic variant in driving causal inference.^[31–33]^ To mitigate the incidence of false positives attributable to random effects, we implemented the Bonferroni correction method for multiple comparisons. The significance threshold established for Bonferroni-adjusted multiple testing was *P* < 2.62 × 10⁻⁴ (calculated as 0.05/191, where 0.05 is the significance threshold for individual tests, 191 means the total number of rsfMRI phenotypes in the study).^[34]^

## 3. Result

**Figure 2.**
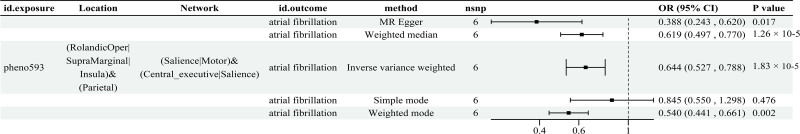
Forest plot for the causal effect of rsfMRI phenotype on the risk of atrial fibrillation derived from inverse variance weighted (IVW), weighted median, MR Egger, Simple mode, and Weighted mode. CI = confidence interval, MR = Mendelian randomization, OR = odds ratio, rsfMRI = resting-state functional magnetic resonance imaging.

**Figure 3.**
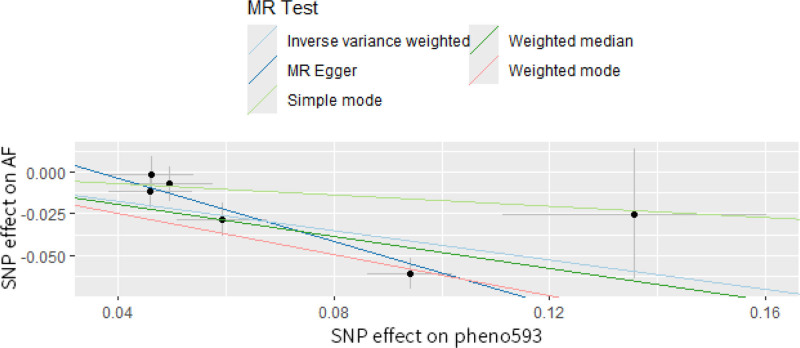
Scatter plot for estimating the change in rsfMRI phenotype on the risk of atrial fibrillation. rsfMRI = resting-state functional magnetic resonance imaging.

**Figure 4.**
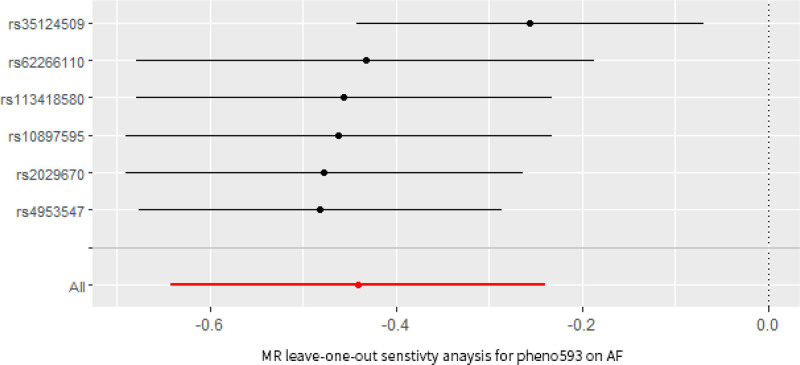
Leave-one-out analysis for rsfMRI phenotype on atrial fibrillation. rsfMRI = resting-state functional magnetic resonance imaging.

In our MR analysis, FC between the precuneus, angular gyrus, cingulate gyrus, and parietal lobe was negatively correlated with the risk of developing atrial fibrillation (AF), and activity in these brain regions influenced the FC between salience network (SN), default mode network (DMN), and Central Executive Network (CEN) (OR_IVW_ = 0.644, 95% CI: 0.527–0.788, *P* = 1.83 × 10^‐5^). Additionally, we determined a causal relationship between AF and a rsfMRI phenotype associated with FC of the triple network as well as the attention network. Specifically, a 1-standard-deviation rise in global measured FC was associated with a 15.7% reduction in AF risk (OR_IVW_ = 0.843, 95% CI: 0.779–0.912, *P* = 2.17 × 10^‐5^). The *F*-statistics for all IVs used in this study exceeded 10, indicating the validity and robustness of IV (Table S2, Supplemental Digital Content, https://links.lww.com/MD/P312). Using Cochran Q test, Global test, and MR-Egger intercept method, our results suggest that the causal relationship between network FC of the brain as a whole and AF is affected by tests of horizontal pleiotropy, which contradicts the 3 main assumptions of MR analysis. The results of our final MR analysis are shown in Fig. 2. FC of brain networks between the precuneus, angular gyrus, cingulate gyrus, and parietal lobe is unaffected by heterogeneity and horizontal pleiotropy (Table 2). The scatter plots of the MR analysis are shown in Fig. 3. The results of the leave-one-out method showed that the overall effect value and confidence interval at all SNPs were (*β* ± SE: ‐0.440 ± 0.103, *P* = 1.83 × 10^‐5^). Table S3, Supplemental Digital Content, https://links.lww.com/MD/P313 shows each SNP exclusion’s effect value and confidence interval. Our results suggest the consistency of the results of the leave-one-out analyses, with none of the SNPs substantially altering the conclusions, which supports the robustness of our findings (Fig. 4). In addition, we found no causal relationship between functional brain networks and other CVDs.

**Table 2:** Heterogeneity and horizontal pleiotropic test between functional connectivity of brain networks and cardiovascular disease.

Exposure	Outcome	Cochran Q statistic	*P*-value for Cochran Q	Egger intercept	*P*-value for Egger intercept	*P*-value for global test
pheno593	Atrial fibrillation	11.011	.051	0.034	.090	.131
pheno1698	Atrial fibrillation	38.972	.020	0.020	.010	.011

## 4. Discussion

We employed a two-sample MR analysis to elucidate the causal relationships between 191 brain resting-state fMRI phenotypes and 10 CVDs. Our primary findings revealed that decreased FC within the DMN, SN, and CEN (specifically involving the precuneus, angular gyrus, cingulate gyrus, and parietal lobe) was significantly associated with an elevated risk of AF (OR_IVW_ = 0.644, 95% CI: 0.527–0.788, *P* = 1.83 × 10^‐5^).

The interplay between the heart and the brain is now the focus of increasing interest. The heart-brain axis is defined as the physiological interactions between the circulatory and nervous systems, which are characterized by bidirectional communication via autonomic nerves, hormones, and cytokines.^[35]^ This intricate network is implicated in the pathogenesis of an assortment of CVDs, including hypertension, congestive HF, and AF.^[36]^ In instances of hypertension and HF, pro-inflammatory cytokines within the central nervous system can modulate sympathetic outflow, consequently contributing to cardiac remodeling.^[35]^ Additionally, neurological dysregulation may impair cardiac function, as evidenced by stress-induced cardiac dysfunction (takotsubo cardiomyopathy) and stroke-associated cardiac syndrome.^[37]^ Conversely, cardiac dysregulation has the potential to negatively impact neurological function, as evidenced by arrhythmia-related neurological complications, myocardial ischemia-associated neurological issues, and systemic microvascular damage.^[38–40]^

Brain network function refers to the complex interconnections of nerve fibers between various brain regions, which regulate cognitive, behavioral, and emotional processes.^[41]^ This concept encompasses the interactions and information transfer among distinct brain areas that collaborate to perform specific tasks. Research on brain network function commonly utilizes neuroimaging techniques, such as fMRI, to analyze brain activity patterns in a resting state.^[10]^ The DMN is proactive during rest and is involved in self-referential thinking, introspection, and memory recall. The CEN supports higher cognitive functions, including decision-making, problem-solving, and attentional control. The SN is responsible for rapid responses to environmental stimuli and attention shifting. These 3 networks form the core of brain network function and are intricately interconnected, collectively supporting the brain’s complex operations.^[42]^ The precuneus, located in the prefrontal lobe, plays a critical role in decision-making, emotion regulation, behavioral control, and self-monitoring.^[43]^ The angular gyrus, situated in the posterior parietal lobe, is integral to various cognitive functions, including language processing, mathematical reasoning, spatial awareness, and emotional cognition. The cingulate gyrus, a key component of the limbic system, is closely associated with emotional processing, pain perception, learning, and memory.^[44]^ Additionally, the parietal lobe is significantly involved in the regulation of mood, stress response, cognitive functions, and autonomic control, further underscoring its broad influence on both emotional and physiological states.^[45]^ Changes in these brain regions are closely linked to the regulation of emotions, stress, and stress response, potentially increasing the risk of AF by disrupting autonomic homeostasis. Current evidence indicates a significant alteration in sympathetic nerve activity in patients with AF relative to healthy individuals, particularly with an increase in muscle sympathetic nerve activity under conditions of rapid atrial pacing.^[46]^ The DMN, comprising the precuneus, medial prefrontal cortex, and posterior cingulate cortex, plays a role in regulating muscle sympathetic nerve activity. In a cross-sectional study of AF patients without a history of stroke, a notable reduction in DMN FC was observed in comparison to healthy controls, suggesting a potential correlation between AF and DMN connectivity.^[47]^ In a prospective cohort study of 150 patients with acute ischemic stroke (56 with right-hemisphere lesions and 94 with left-hemisphere lesions), severe arrhythmias were observed in 49 cases, corresponding to a prevalence of 32.7%. Voxel-based lesion-symptom mapping analysis demonstrated a significant association between lesions in specific brain regions and the occurrence of severe arrhythmias (false discovery rate-corrected, *P* < .05). The affected brain regions included the right insular cortex, right frontal cortex, right parietal cortex, right amygdala, basal ganglia, and thalamus.^[48]^ In a prospective cohort study involving 1661 first-time stroke patients, 41 were identified with newly-onset AF at admission or within the first 3 days poststroke. The study also found that parietal insula lesions (32%) and brainstem lesions (11%) were more common in patients with recent AF (16.7% and 6.7%, respectively) compared to those with cardioembolic stroke. This suggests that these brain regions may be more vulnerable to the effects of AF following a stroke.^[49]^

The central autonomic nervous system controls cardiovascular function through both pre-sympathetic and parasympathetic pathways, which together form the exogenous cardiac nervous system. Key brain regions, including the prefrontal cortex, cingulate cortex, amygdala, and hippocampus, modulate sympathetic inputs to the heart, thus regulating cardiovascular function. The intrinsic cardiac autonomic nervous system is made up of ganglionic plexuses along the pulmonary veins in the left atrium and within the pericardium, significantly modulated by the peripheral autonomic nervous system, particularly in the insula. Dysregulation of the sympathetic and parasympathetic systems, often resulting from insular infarction, can lead to AF.^[50–52]^ Insular structural alterations are linked to chronic inflammatory responses, including microglial activation and the liberation of cytokines such as tumor necrosis factor-α and interleukin-6. In the acute phase of a stroke, brain damage elicits an inflammatory response characterized by microglial and astrocytic proliferation, resulting in significant cytokine and chemokine release. Simultaneously, endothelial damage compromises the blood–brain barrier, allowing pro-inflammatory molecules to enter peripheral circulation. Over time, inflammatory mediators progressively influence atrial electrophysiology and structural components via multiple signaling pathways, thereby heightening the risk of AF.^[53–55]^ Our study findings reveal that network FC in the precuneus, angular gyrus, cingulate gyrus, and parietal lobe regions is negatively correlated with AF. The interaction of these brain regions is engaged in the regulation of the autonomic nervous system, and autonomic dysregulation is considered an important risk factor for AF.

Our findings suggest that the DMN and the SN, especially the reduced FC involving the precuneus gyrus, angular gyrus, and cingulate gyrus, may serve as novel biomarkers for risk stratification of AF. The dysfunction of these brain regions, as key neural hubs for autonomic regulation and stress response, may reflect an early state of neural compensatory imbalance before the onset of AF.^[56]^ Compared with the sensitivity limitations of conventional electrocardiography in the detection of subclinical AF, rsfMRI, with its high spatiotemporal resolution, can quantitatively assess the dynamic reorganization of neural networks, capture subtle functional abnormalities before structural damage or clinical symptoms become apparent, and provide a critical time window for early warning of AF risk. Multimodal integration strategies (e.g., combining rsfMRI with ambulatory electrocardiographic monitoring) can significantly improve the early identification of high-risk individuals presenting with HRV abnormalities, asymptomatic sympathetic hyperactivity, or autonomic dysfunction. In addition, noninvasive neuromodulation techniques (e.g., transcranial magnetic stimulation) can modulate neurological activity in a variety of disease states and have been shown in previous studies to alter arrhythmia risk by targeting cardiac sympathetic innervation.^[57–59]^ Such noninvasive interventions offer a new direction for the precise treatment of AF associated with autonomic imbalance.

A key strength of our study is the use of rsfMRI for MR analysis. rsfMRI is a functional tool for examining FC in the brain at rest, identifying activity patterns in brain networks, and revealing dynamic interactions between regions, unlike conventional MRI, which primarily focuses on anatomical imaging. Additionally, we employed the Bonferroni-adjusted method for multiple testing, enhancing the robustness of our conclusions. The Bonferroni correction was applied to mitigate the risk of Type I errors (false positives) given the multiple comparisons. However, this stringent adjustment increases the likelihood of Type II errors (false negatives), potentially obscuring true associations that do not reach the adjusted threshold. For instance, while only the association between brain network connectivity and AF survived Bonferroni correction (*P* < 2.62 × 10⁻⁴), several other phenotypes showed nominally significant associations (*P* < .05) with CVDs, and also, to more flexibly balance the Type I and Type II errors, We applied a false discovery rate control to the results of all IVW methods (Table S4, Supplementary Digital content, https://links.lww.com/MD/P315). These findings necessitate additional investigation through multi-center clinical trials with expanded sample sizes, coupled with mechanistic validation utilizing controlled in vitro assays and in vivo animal models, to corroborate the observed associations and elucidate potential biological pathways. Nevertheless, the Bonferroni approach was chosen for its simplicity and conservatism in maintaining study-wide error control, particularly given the exploratory nature of brain-heart interactions.^[60–62]^

In the present study, we found that the association between FC of brain networks and the rest of CVDs did not reach the Bonferroni correction threshold, except for AF. The pathophysiological mechanisms of AF are closely related to autonomic regulation, and functional changes of brain networks (e.g., the DMN, the CEN) may directly regulate cardiac electrical activity by influencing the hypothalamus–pituitary–adrenal axis and the brainstem autonomic nervous system. Axis and brainstem autonomic nuclei to directly modulate cardiac electrical activity.^[35,36]^ In contrast, the pathogenesis of other CVDs is more dependent on inflammation, abnormalities in lipid metabolism, or vascular endothelial dysfunction, processes that may be less directly regulated by the rsfMRI phenotypes selected for this study.^[63]^

Our study also has certain limitations. First, the GWAS dataset primarily represents a European population, highlighting the need for validation in other populations. Second, the age discrepancy between participants may affect causal inferences regarding age-related cardiovascular conditions, such as HF. Third, despite rigorous IV selection and sensitivity analyses, the lack of individual-level data limited our ability to evaluate other potential confounders (e.g., population stratification), which may introduce bias. Fourth, current MR methods rely on various assumptions. While there is some correlation between genetic variation and environmental influences, genetic variation is improbable to perfectly replicate environmental influences, potentially biasing result interpretations. Fifth, the small sample size of GWAS for several CVDs resulted in insufficient statistical power of IVs. Finally, although our MR analyses suggest a causal relationship between changes in network connectivity and CVD, these findings are primarily based on statistical inference and require validation through further clinical longitudinal studies.

## 5. Conclusion

Our study identified decreased FC among the salience, DMN, and CEN in the Precuneus, angular gyrus, cingulate gyrus, and parietal lobe were also associated with AF. Our study suggests that regular electrocardiograms should be performed early in the population with abnormal magnetic resonance imaging at rest for early detection of patients with cardiac arrhythmias. In addition, transcranial magnetic stimulation may be an effective therapeutic target for these patients. Moreover, no MR analyses of brain network FC associated with other CVDs met the Bonferroni-adjusted threshold (*P* < 2.62 × 10⁻⁴).

## Acknowledgments

Thanks to all authors for their contributions and to the Zenodo database and the FinnGen consortium for providing data.

## Author contributions

**Conceptualization:** Yushi Wang.

**Investigation:** Shipeng Wang.

**Methodology:** Han Chi Xu.

**Software:** Zhen Guo, Yulin Tian.

**Writing – original draft:** Haoxuan Chu, Xia Guo.

## Supplementary Material


